# A comprehensive phylogeography of the widespread pond snail genus *Radix* revealed restricted colonization due to niche conservatism

**DOI:** 10.1002/ece3.8434

**Published:** 2021-12-17

**Authors:** Takumi Saito, Takahiro Hirano, Bin Ye, Larisa Prozorova, Mohammad Shariar Shovon, Tu Van Do, Kazuki Kimura, Purevdorj Surenkhorloo, Yuichi Kameda, Yuta Morii, Hiroshi Fukuda, Satoshi Chiba

**Affiliations:** ^1^ Graduate School of Life Science Tohoku University Sendai Japan; ^2^ Department of Biology Faculty of Science Toho University Funabashi Japan; ^3^ Center for Northeast Asian Studies Tohoku University Sendai Japan; ^4^ Federal Scientific Center of the East Asia Terrestrial Biodiversity Far Eastern Branch Russian Academy of Sciences Vladivostok Russia; ^5^ Department of Biochemistry and Molecular Biology University of Rajshahi Rajshahi Bangladesh; ^6^ Institute of Ecology and Biological Resources Vietnam Academy of Science and Technology Ha Noi Vietnam; ^7^ Graduate University of Science and Technology Vietnam Academy of Science and Technology Ha Noi Vietnam; ^8^ Department of Biology Research Institute for Ulleung‐do and Dok‐do Islands Kyungpook National University Daegu Korea; ^9^ Mongolian Benthological Society‐MOBS Ulaanbaatar Mongolia; ^10^ Center for Molecular Biodiversity Research National Museum of Nature and Science Tsukuba Japan; ^11^ Laboratory of Animal Ecology Department of Zoology Graduate School of Science Kyoto University Kyoto Japan; ^12^ The Hakubi Center Kyoto University Kyoto Japan; ^13^ Conservation of Aquatic Biodiversity Faculty of Agriculture Okayama University Okayama Japan

**Keywords:** dispersal, freshwater snail, latitudinal diversity gradient, Lymnaeidae, Mollusca, passive disperser, tropical conservatism hypothesis

## Abstract

To clarify the effect of niche conservatism on evolutionary history, we focused on freshwater snails, which have different ecological and phylogenetic properties from previously tested taxa. We conducted a phylogenetic analysis using 750 lymnaeid individuals from 357 sites of eleven *Radix* species. Then, we estimated the ancestral distribution using the geographic coordinates and colonization routes. In addition, a statistical test of the colonization distances in the latitudinal and longitudinal directions was performed. We also conducted ecological niche modeling for two widely distributed species using climatic data. Ancestral geographic reconstruction estimated the origin of the genus to be around the Indian subcontinental region and showed that latitudinal immigration distances were shorter than longitudinal immigration distances in the diversification process. Ecological niche models suggested that the current distribution was restricted by climate, with annual mean temperature and precipitation of the driest month as particularly strong factors. Niche conservatism to the climate can affect the diversification of freshwater snails.

## Graphical abstract

To clarify the effect of niche conservatism on evolutionary history, we conducted a comprehensive phylogeography and estimation of dispersal routes. As a results, niche conservatism can affect the diversification of freshwater snails, genus *Radix*.

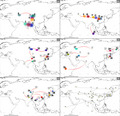

## 1 INTRODUCTION

Whether or not ecological traits have restricted current biodiversity patterns is a critical issue. In particular, niche conservatism (NC), namely the retention of niche‐related ecological traits through evolutionary history (see Wiens et al., 2010), is an attractive theme that bridges ecology and evolutionary biology (Peterson et al., 1999; Wiens et al., 2010; Wiens & Donoghue, 2004; Wiens & Graham, 2005). In a biogeographical context, NC, especially as a process that leads to current biodiversity patterns, is often suggested to be important in limiting distribution transitions (Losos, 2008; Pearman et al., 2008; Wiens & Graham, 2005). In particular, NC could explain the latitudinal diversity gradient (LDG), which is one of the principal themes of historical biogeography (Fine, 2015; Hillebrand, 2004; Mannion et al., 2014; Mittelbach et al., 2007; Pontarp et al., 2019). In the context of the LDG, NC is often recognized as the basis of the tropical conservatism hypothesis (TCH; Pianka, 1966; Wiens & Donoghue, 2004; Wiens & Graham, 2005). Organisms in tropical regions that occupied greater areas in the past (~34 Ma; Bowen et al., 2015; Cronin, 2010) have restricted evolutionary transitions over different climatic regions due to NC, leading to the LDG (Farrell et al., 1992; Peterson et al., 1999; Wiens & Donoghue, 2004). Recently, the TCH has been evaluated as a potential driver of the LDG in many phylogeographic studies (e.g., Buckley et al., 2010; Duchêne & Cardillo, 2015; Economo et al., 2018, 2019; Kerkhoff et al., 2014; Owens et al., 2017). Moreover, NC can also drive diversity gradients from a species origin outside the tropics, and NC has universal importance for explaining biodiversity patterns (Wiens et al., 2010). In fact, some biogeographic studies have shown a peculiar diversity gradient pattern in some taxa caused by NC (i.e., the highest diversities occur outside of the tropics) (e.g., Morales‐Castilla et al., 2020; Morinière et al., 2016; Pyron & Burbrink, 2009; Quintero & Jetz, 2018; Stephens & Wiens, 2009). Furthermore, NC can explain other diversity patterns (Wiens et al., 2010). For example, the high diversity seen at mid‐elevations is understood to be a result of NC (e.g., Kozak & Wiens, 2010; Li et al., 2009; Szewczyk, & McCain, 2019).

In contrast to the clear evidence for the contribution of NC to the diversity patterns of many taxa, the patterns of some taxa are often explained by other mechanisms such as competition, carrying capacities, and the places of the origin of organisms (Pyron & Wiens, 2013; Ramos Pereira & Palmeirim, 2013; Rolland et al., 2014; Siqueira et al., 2016). More importantly, studies comparing a large number of taxonomic groups have concluded that the contribution of NC to the LDG was fairly limited (Boucher‐Lalonde et al., 2015; Jansson et al., 2013). Although phylogenetic studies are useful in understanding the importance of NC (Wiens et al., 2010; Wiens & Donoghue, 2004; Wiens & Graham, 2005), most of these studies have been conducted with vertebrates, plants, and insects due to limited taxon sampling (Fine, 2015; Jablonski et al., 2017). However, as ecological differences such as the dispersal mode of each taxon can interactively alter the effect of NC on the evolutionary process (Ackerly, 2003; Eiserhardt et al., 2013; Kubota et al., 2017), the relative importance of NC in evolutionary history can differ depending on the taxon considered (Chiu et al., 2020). While further studies on various taxa with different ecological traits may be useful (Chiu et al., 2020), the importance and contribution of NC to the diversification process are poorly understood, except for some taxa (e.g., Amphibians: Kozak & Wiens, 2010; Reptiles: Pyron & Burbrink, 2009; Stephens & Wiens, 2009).

Here, we focused on the common freshwater snail genus *Radix* to address this issue. *Radix* belongs to the family Lymnaeidae (Gastropoda) and has a wide distribution range covering most of the Old World, from the tropics to the subarctic regions (Aksenova et al., 2018). In addition, *Radix* species has high morphological diversity including phenotypic plasticity in their shell (Pfenninger et al., 2006; Terry & Duda, 2021; Ward et al., 1997), and thus many morphological species have been described (Vinarski et al., 2020). However, many species are currently considered to be synonyms, and nine species and one undescribed species have been detected based on the molecular taxonomy (Aksenova et al., 2018). In general, freshwater snails are often dispersed by water currents, and can rarely be transported by wind and animals such as birds, amphibians, insects, and mammals (Bespalaya et al., 2020; Kappes & Haase, 2012; van Leeuwen et al., 2012; Rees, 1965; Walther et al., 2008). As a result, they have low active and high passive dispersal potential (Kappes & Haase, 2012), and often have a wide distribution range. Perhaps as a consequence, freshwater snails seem to have relatively high niche flexibility, including niche shifts within the species (Cordellier & Pfenninger, 2008, 2009; Kisdi, 2002; Torres et al., 2018). These ecological characteristics may mean that colonization is unlikely to be strongly hindered by causes other than climatic factors (e.g., the significant lack of dispersal ability or the habitat dependence on interspecies relationships such as symbiosis) and may suggest that the distribution transitions of freshwater snails could be based on more purely probabilistic processes than that of well‐studied vertebrates having low dispersal ability such as amphibians and reptiles. This is quite different from the taxa traditionally used to examine NC and diversification. The effects of NC on the historical diversification of freshwater snails have been largely unexamined; therefore, the verification of the effect of NC using freshwater snails could provide new insights into the contribution of NC to diversification.

In this study, we collected comprehensive *Radix* materials and sequences from their whole distribution area and conducted phylogenetic analyses to estimate the historical distribution transition. We took into account the phylogeographical structure within each species, which many conventional NC studies using phylogenetic approaches have not fully addressed. In addition, we quantitatively evaluated the latitudinal and longitudinal (L/L) dispersal for each distribution transition event by reconstructing the historical distribution change under specific latitudes and longitudes. The distribution transitions of each lineage have previously been evaluated as either categorical data or indicator values (e.g., Duchêne & Cardillo, 2015; Kerkhoff et al., 2014). These evaluations do not adequately assess colonization within the same climate zone, even though the climate is continuous. In contrast, our approach may be able to accurately estimate the L/L dispersal distances for each colonization event within the same climate zone. In other words, more colonization events are expected to have a longer dispersal distance in the longitudinal direction than in the latitudinal direction if the adaptation to the new niches of climatic temperature restricts the distribution transition. Furthermore, we also performed ecological niche modeling to identify the climatic characteristics restricting the colonizations. Finally, we integrated these approaches and validated the contribution of NC as a process, focusing on the transitions in the distribution area.

## 2 METHODS

### 2.1 Sampling and DNA methods

**FIGURE 1:**
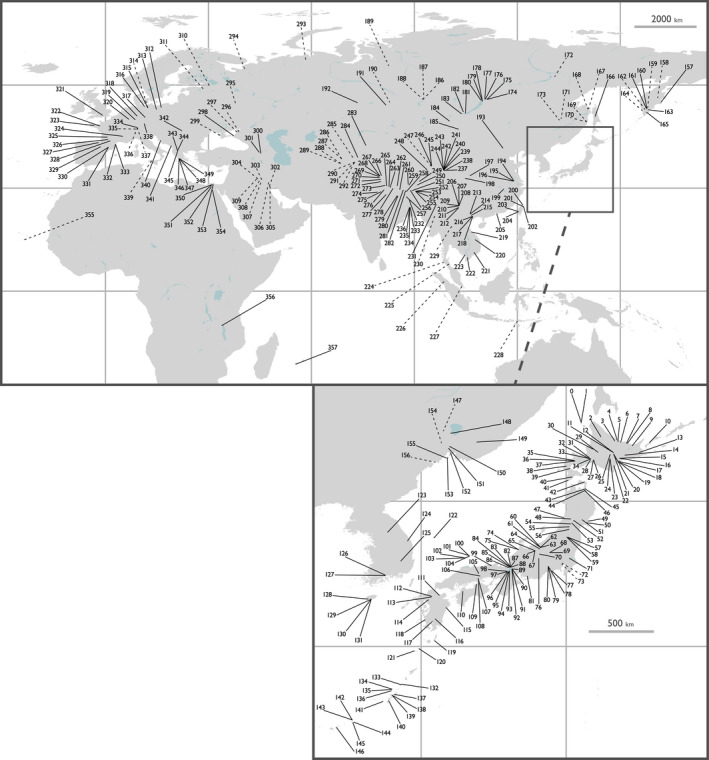
The map of localities where the materials of examined *Radix* spp. were collected. Solid lines denote sites obtained: the latitudinal and longitudinal (L/L) information from sampling data, published references, or Genbank registration data. Dashed lines denote sites estimated L/L information based on given locality names. This map was generated from Global Self‐consistent, Hierarchical, High‐resolution Geography Database, Version 2.3.5 (Wessel & Smith, 1996, 2016), using QGIS Version 2.18 (QGIS Development Team, 2016). See Table S1 for further information

We collected data from 750 lymnaeid individuals from 357 sites across nine countries and regions—278 of the individuals were sampled by us and the remaining data were obtained from GenBank (Figure 1 and Table S1). The L/L information of samples from GenBank was identified using published papers and registered information from GenBank (Table S1). When L/L information was not available, it was assigned based on the location name of the sampling site (e.g., the specific lake, village, or township) (Figure 1). This could be done accurately for most samples, as detailed site names were usually provided. These materials covered the greater part of the *Radix* distribution area.

Total DNA was isolated from the samples using a DNeasy Blood & Tissue Kit (Qiagen) according to the manufacturer's instructions. We sequenced fragments of the mitochondrial cytochrome c oxidase subunit 1 (CO1), internal transcribed spacer 2 (ITS2), 28S ribosomal RNA (28S), and Histone 3 (H3) (Table S1). The polymerase chain reaction (PCR) protocol and primers are detailed in Table S2. The PCR products were purified with Exo‐SAP‐IT (Amersham Biosciences). Sequencing was performed using a BigDye™ Terminator Cycle Sequencing Ready Reaction Kit (Applied Biosystems) and electrophoresed using an ABI 3130xl sequencer (Applied Biosystems). We obtained 278 CO1, 66 ITS2, 77 28S, and 80 H3 sequences. The obtained CO1, ITS2, 28S, and H3 sequences were deposited in the GenBank database (Table S1).

### 2.2 Phylogenetic methods

We conducted a phylogenetic analysis using CO1 sequences (278 newly collected by us and 472 from GenBank). Eight species were selected as outgroups from the eight most closely related genera with reference to a previous phylogenetic study (Aksenova et al., 2018). These sequences were aligned with MUSCLE v3.8 (Edgar, 2004), and there was no gap in the alignment. CO1 phylogenetic trees were determined using both the Bayesian inference (BI) and maximum likelihood (ML) methods. Before both analyses, the same sequences were stacked using FaBox1.41 (Villesen, 2007), and as a result, 455 haplotypes were detected in the genus *Radix* (Table S1). Next, we selected the appropriate partitioned models of sequence evolution using PartitionFinder2 (Lanfear et al., 2016; Table S3). Based on these models, the BI analysis was performed using MrBayes5d version 3.1.2.2012.12.13 (Tanabe, 2012a), an extended software of MrBayes v3.1.2 (Ronquist & Huelsenbeck, 2003), with two simultaneous runs. We discarded non‐convergence trees after examining convergence and effective sample size (ESS; larger than 200) using Tracer v. 1.6 (Rambaut et al., 2013) and the remaining samples were used to estimate phylogeny (for detailed settings, see Table S3). ML analysis was performed using IQ‐TREE version 1.6.7 (Nguyen et al., 2015) and the evolutionary model was selected for IQ‐TREE with the –spp option (for detailed settings and the model, see Table S3). For the ML analysis, we assessed nodal support by performing ultrafast bootstrapping (Hoang et al., 2018) with 5000 replications. These phylogenetic analyses were partly assisted by Phylogears2 version 2.0.2012.02.13 (Tanabe, 2012b) as a pipeline for sequence files. Furthermore, the distribution area of each species was calculated and illustrated using QGIS (QGIS Development Team, 2016) based on the samples used in the phylogenetic analysis of CO1.

We reconstructed both nuclear (H3+ITS2+28S) and combined (CO1+H3+ITS2+28S) phylogenies to assess phylogenetic positions among each species estimated by CO1 phylogeny. We used 81 individuals from 9 *Radix* species and 83 individuals from 11 *Radix* species for nuclear phylogeny and combined phylogeny, respectively (Table S1). *Racesina luteola* was selected as an outgroup for both phylogenies. To eliminate the uncertainty of the ITS2 and 28S alignments, trimAl 1.2 (Capella‐Gutiérrez et al., 2009) was used for subsequent phylogenetic analyses. All phylogenetic analyses were performed using the same approach as that used for the CO1 phylogeny (for detailed settings, see Table S3).

### 2.3 Geographic reconstruction

To trace the historical changes in the distribution of taxa, we performed Bayesian ancestral distribution reconstruction using BayesTraits v.3.0.1 under a geographical model (Pagel, 2004, 2017). A Bayesian CO1 tree was used after removing outgroups and using L/L information (Table S1). The branch lengths were scaled to have a mean of 0.1 with reference to the BayesTraitsV3 manual (Maede & Pagel, 2016). Then, reconstruction was estimated under the following settings: ngen = 15,000,000, sample freq = 1000, burn‐in = 5,000,000, and rate dev = autotune. Then, the estimated L/L sites on each principal node were denoted using QGIS. Furthermore, to evaluate historical L/L colonization, the L/L immigration distances between pairs of sites in the sequenced nodes were calculated using the GRS80 model. Finally, we conducted an exact Wilcoxon signed‐rank test to examine the difference between the obtained distances of L/L immigration using the package “exactRankTests” in R version 3. 5. 1 (R Core Team, 2018; Torsten & Kurt, 2019).

### 2.4 Ecological niche modeling

To clarify whether climatic variables with latitudinal gradients actually restrict the *Radix* distribution, we conducted ecological niche modeling (ENM) using MaxEnt version 3.4.1 (Phillips et al., 2004, 2006, 2017). We performed ENM with two species with a relatively large sample size, *R. auricularia* and *R*. *plicatula*. The potentially dispersible landmass of the species was presumed to be −20 to 180° latitude and 0–90° longitude. First, 19 bioclimatic variables with a spatial resolution of 2.5 arcmin were obtained from WorldClim 2 (Fick & Hijmans, 2017), as proxies for the ecological traits of *Radi*x. To obtain a parsimonious and interpretable model (Merow et al., 2013), we eliminated spatially correlated bioclimatic variables of current climate data prior to the modeling using ENMTools (Warren et al., 2010, 2019) in R (R Core Team, 2018). As MaxEnt is relatively robust for high collinearity (Elith et al., 2011), we only removed the variable pair with a correlation coefficient greater than |0.80|. In the modeling process, we used only linear and quadratic features, and the regularization multiplier was set to 3 to avoid overfitting (Merow et al., 2013; Radosavljevic & Anderson, 2014; Syfert et al., 2013). In the pilot analyses, this setting had little effect on the area under the receiver operating characteristic (ROC) curve, which represents the predictive accuracy. MaxEnt runs were conducted under the following settings: maximum number of background points = 10,000, duplicate presence records = remove, maximum iterations = 5000, and output format = Cloglog. Models were evaluated using fivefold cross‐validation and the AUC as an indicator. Moreover, to determine which environmental variables were important drivers of the distribution, we used three different approaches (Phillips, 2017). First, we determined the percent contribution based on the increase or decrease in regularized gain under model construction with MaxEnt. Second, we used permutation importance based on the drop in training AUC when the values of that variable on training presence and background data were randomly permuted for each variable. Third, we used the jackknife test, that is, each variable was deleted sequentially and a model was created with the remaining variables. Next, a model was created with each variable in isolation and each corresponding model was compared. These approaches are considered popular metrics for the evaluation of variable contributions (Bradie & Leung, 2016).

## 3 RESULTS

### 3.1 Phylogenetic relationship of *Radix*

**FIGURE 2:**
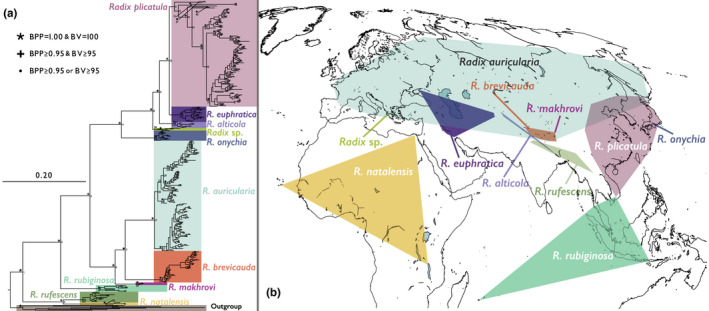
(a) The Bayesian phylogenetic tree of the *Radix* inferred from 455 CO1 haplotypes from 750 sequences (660 bp). Eight species were selected as outgroups. Scale bar indicates substitutions per site. Numbers on the branches represent principal node numbers for convenience. Marks on the branches indicate the Bayesian posterior probabilities (BPP) and the Maximum likelihood ultrafast bootstrapping value by IQ‐TREE (Hoang et al., 2018; Nguyen et al., 2015). In this tree, these values of relatively well‐supported branches are only shown. See Table S1 for further information. Each *Radix* species was determined based on a previous study (Aksenova et al., 2018). (b) The map of distribution areas of each *Radix* species. This map is illustrated in the Mollweide projection to show the correct area. This map is generated from Global Self‐consistent, Hierarchical, High‐resolution Geography Database, Version 2.3.5 (Wessel & Smith, 1996, 2016), using QGIS Version 2.18 (QGIS Development Team, 2016). For further information, see also Figure 1 and Table S1

**FIGURE 3:**
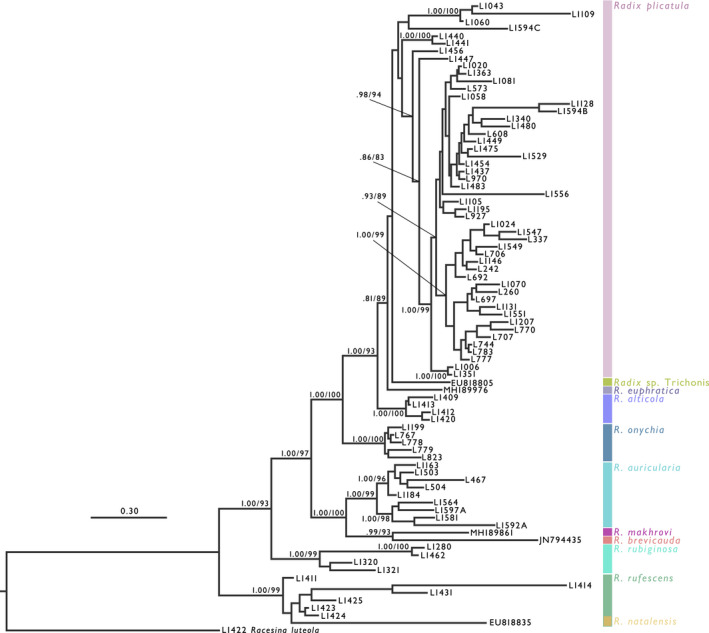
The Bayesian phylogenetic tree of the *Radix* inferred from the combined dataset (1945 bp). *Racesina luteola* was selected as an outgroup. Each operational taxonomic unit label represents the material ID. Scale bar indicates substitutions per site. Numbers on the branches indicate the Bayesian posterior probabilities (BPP) and the Maximum likelihood ultrafast bootstrapping value by IQ‐TREE (Hoang et al., 2018; Nguyen et al., 2015). These values of low supported (BPP < 0.070) and terminal branches are not shown. See Table S1 for further information

Our mitochondrial phylogenetic analysis included 11 species (Figure 2a and Supporting Information 4 as Figure S4.1). Seven of the 11 species were fully supported (Bayesian posterior probability [BPP] = 1.00/ML ultrafast bootstrap value [BV] = 100) monophyletic in both BI and ML phylogenies. *Radix euphratica* was well supported (BPP = 1.00/BV = 99) monophyletic, and *Radix* sp. was relatively supported (BPP = 0.86/BV = 100) monophyletic. The monophylies of *R*. *plicatula* and *R. rufescens* were not well supported but can be recognized as paraphyletic species. In addition, the topologies of both the combined tree and nuclear tree were similar to those of the CO1 tree, except for the low‐supported branches (Figure 3). However, the resolution of the nuclear tree was not enough to distinguish most of the species (Figure S4.2). Despite the difference in sample numbers among each species, *R. auricularia* had the widest distribution area, whereas other species had limited distribution areas (Figure 1b and Table 1).

**TABLE 1:** Information of current distribution area of *Radix* species

Species	Distribution area (km^2^)	Distribution area without the ocean (km^2^)
*Radix auricularia*	3.37 × 10^7^	3.00 × 10^7^
*R. natalensis*	1.34 × 10^7^	1.29 × 10^7^
*R. rubiginosa*	9.90 × 10^6^	1.16 × 10^6^
*R. plicatula*	7.24 × 10^6^	4.07 × 10^6^
*R. euphratica*	2.39 × 10^6^	2.36 × 10^6^
*R. rufescens*	6.38 × 10^5^	6.38 × 10^5^
*R. brevicauda*	3.66 × 10^5^	3.66 × 10^5^
*R. alticola*	1.35 × 10^5^	1.35 × 10^5^
*R. onychia*	7.22 × 10^2^	7.22 × 10^2^
*Radix* sp. (Lake Trichonis)	4.26 × 10^1^	4.26 × 10^1^
*R. makhrovi*	NA	NA

### 3.2 Geographic reconstruction

**FIGURE 4:**
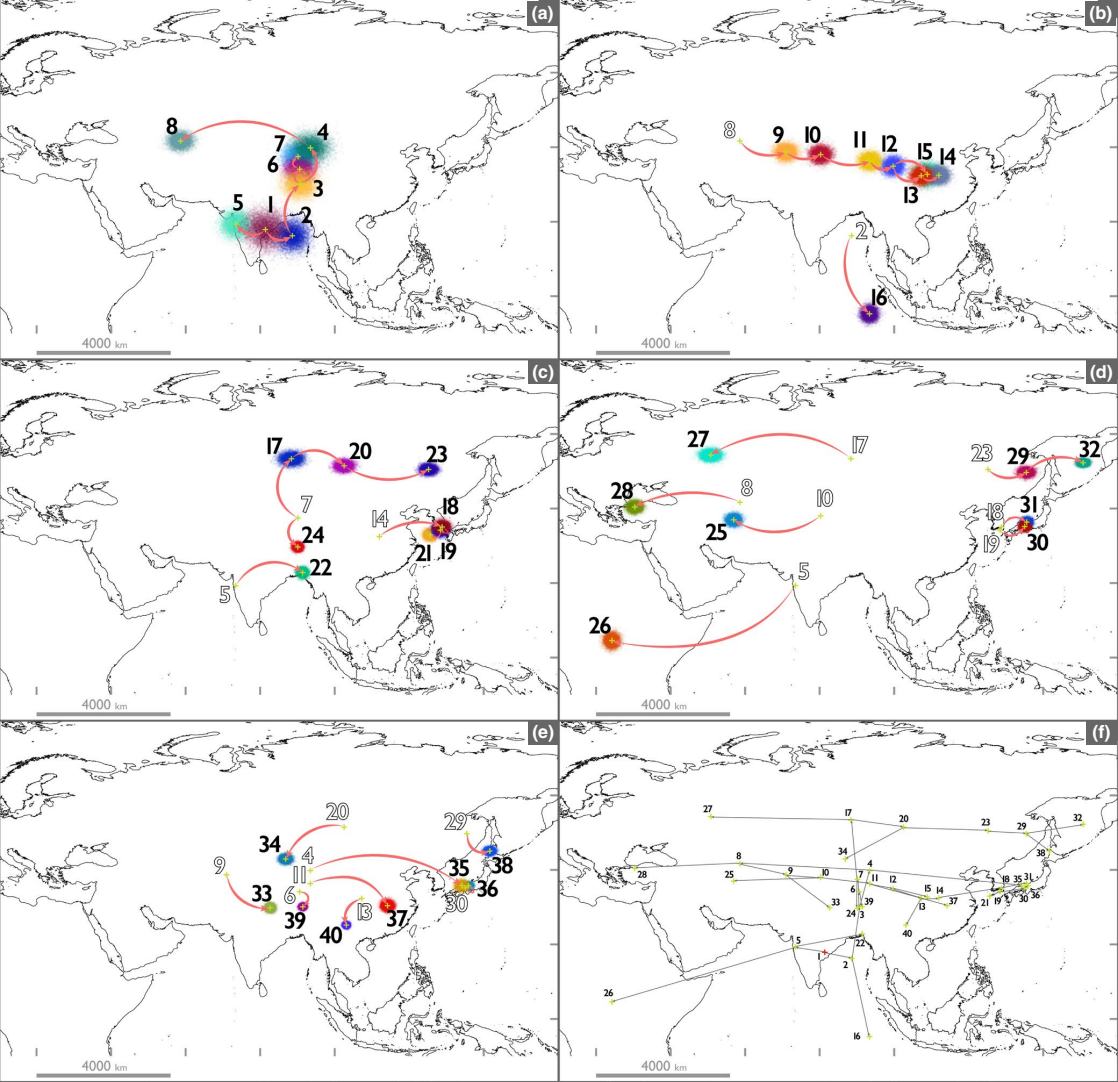
Maps of estimated colonization route of the *Radix*. Numbers on each map represent numbers of the principal node of CO1 phylogeny. These are numbered in order from the primitive branch. Colored areas indicate the estimated past location of each node using BayesTraits v.3.0.1, and its colors are for discrimination. All 10,000 points in each sampled Bayesian generation are illustrated by QGIS under 95% transmittance. The concentrated area with a lot of points indicates the high existence possibility as estimated past location. Crosshairs represent the mean location of the estimated node. Arrows show colonization route on two sequenced nodes. (a) The map of estimated colonization routes in nodes 1–8. (b) The map of estimated colonization route in nodes 9–16. (c) The map of estimated colonization route in nodes 17–24. (d) The map of estimated dispersal route in nodes 25–32. (e) The map of estimated colonization route in nodes 33–40. (f) The summarized map of estimated colonization route in all nodes. The red cross represents the origin of the *Radix*

Our geographic reconstruction estimated the ancestral locations of each node (Table 2). The results suggest that the origin of *Radix* was located around 17.89601652 N, 81.37318393 E (Figure 4a). Furthermore, 39 colonization routes were estimated between the sequenced principal nodes (Figure 4). The calculated L/L immigration distances are shown in Table 3. The longitudinal distances of estimated colonization routes ranged from 1016.323 m to 5,153,093.327 m, with a mean of 1,052,254.515 m, and the latitudinal distances of estimated colonization routes ranged from 14,935.321 m to 2,318,437.031 m, with a mean of 484,870.8215 m. Fifteen of the longitudinal distances and five of the latitudinal distances exceeded 1,000,000 m. There were significant differences among the L/L distances (*p* = .0039).

**TABLE 2:** Information of estimated location of each node

Node no.	Estimated mean latitude and longitude
1	17.89601652/81.37318393
2	16.23072361/88.59765338
3	30.09542406/90.65178895
4	39.81591333/93.47087601
5	19.31821844/73.46168335
6	34.12227066/90.59445368
7	37.47339173/90.13656149
8	41.6728143/58.5940236
9	38.68411615/70.96487848
10	38.03450494/80.26127534
11	36.29365418/93.47426093
12	34.90630679/99.70610308
13	32.33143307/107.2495918
14	32.46611435/111.9989276
15	32.71687105/108.8688617
16	−4.732113464/93.37594267
17	53.35697897/88.39358996
18	35.0642092/128.8174151
19	34.22255405/128.3937568
20	51.4647091/102.5328364
21	32.95448124/125.6583849
22	22.79637394/91.39981877
23	50.50088312/125.1904477
24	29.686688/90.12507201
25	36.98257973/56.93674689
26	4.563551314/24.24970785
27	54.33321132/50.78873303
28	40.42965385/30.38088365
29	49.69964908/135.4618677
30	35.17789691/135.0365486
31	36.36384509/135.5608518
32	52.2719336/150.717363
33	29.8415996/82.69814532
34	42.92299924/86.86289665
35	35.75969895/134.1304039
36	35.69737464/135.8986181
37	30.40400027/114.187158
38	45.07726363/141.6508014
39	30.10905221/91.46695505
40	25.19359861/103.1406435

**TABLE 3:** Information of longitudinal and latitudinal distances of each colonization

Colonization route	Longitudinal distance (m)	Latitudinal distance (m)
1–2	765,506.537	184,297.984
1–5	838,294.041	157,419.920
2–3	219,608.143	1,535,516.385
2–16	510,838.657	2,318,437.031
3–4	271,735.723	1,078,397.895
3–6	5526.767	446,532.666
4–8	2,966,963.557	206,206.968
4–35	3,450,597.144	450,209.719
5–22	1,884,273.032	385,094.516
5–26	5,153,093.327	1,632,270.901
6–7	42,241.530	371,824.846
6–39	80,489.933	445,021.929
7–17	154,175.504	1,765,299.456
7–24	1016.323	863,666.153
8–9	1,029,281.660	331,859.390
8–28	2,338,793.788	138,059.065
9–10	808,534.747	72,108.981
9–33	1,020,216.022	980,882.401
10–11	1,159,006.296	193,200.912
10–25	2,042,271.245	116,750.24
11–12	559,698.625	153,928.442
11–37	1,857,024.702	653,227.851
12–13	689,252.071	285,593.651
12–15	837,111.107	242,850.111
13–14	447,121.179	14,935.321
13–40	386,840.551	791,105.027
14–18	1,579,494.000	288,176.072
17–20	939,881.189	210,562.558
17–27	2,474,537.107	108,656.717
18–19	38,644.726	93,368.229
18–31	614,997.448	144,199.442
19–21	252,038.230	140,648.288
19–30	611,979.280	105,981.061
20–23	1,568,262.743	107,223.657
20–34	1,086,887.423	949,620.158
23–29	728,156.281	89,122.058
29–32	1,098,657.506	286,161.424
29–38	446,352.156	513,909.279
30–36	78,525.799	57,635.333

### 3.3 Ecological niche modeling

**FIGURE 5:**
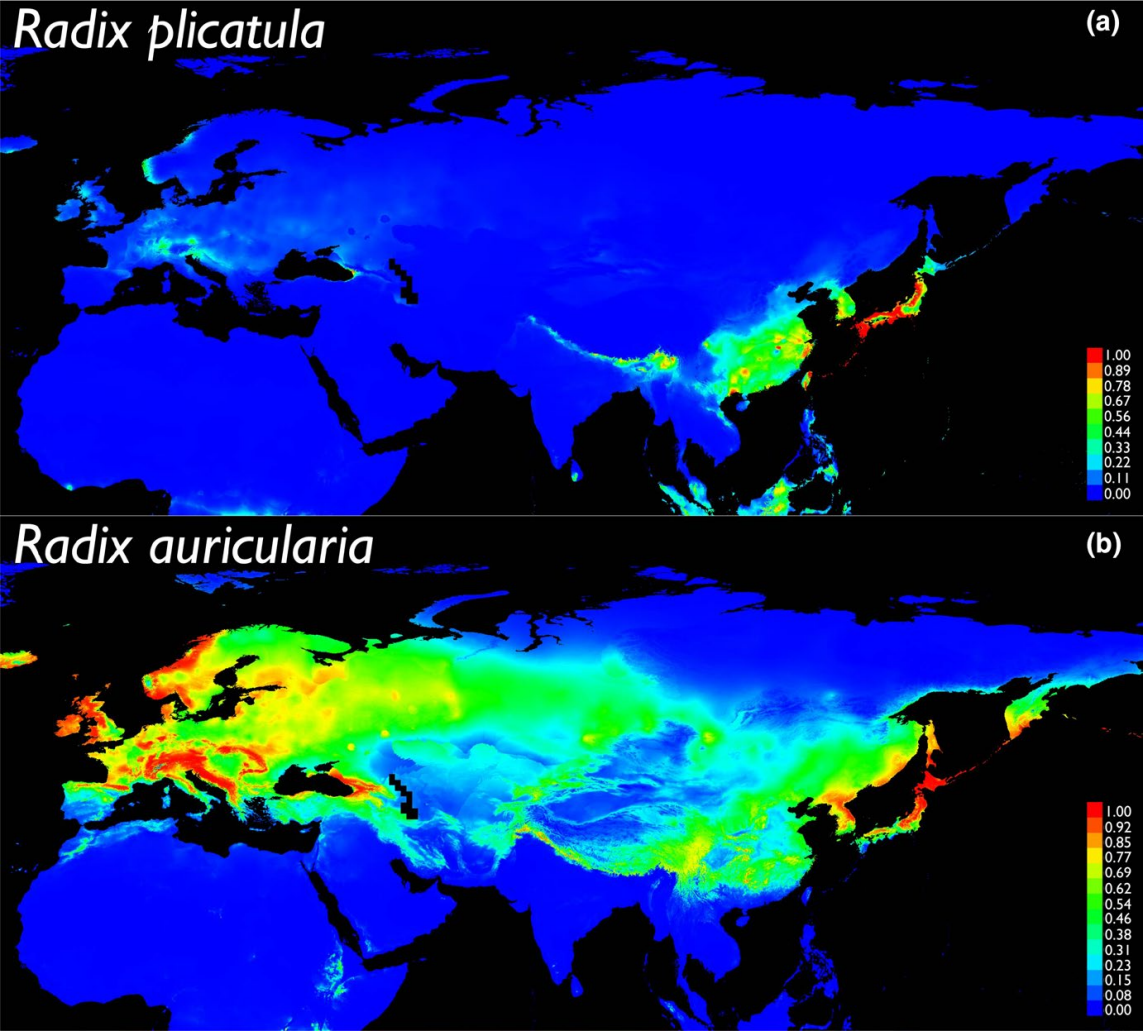
Maps show the occurrence probabilities of two *Radix* species by MaxEnt (Phillips et al., 2004, 2006, 2017). Maps for *Radix plicatula* are shown in a, and maps for *R. auricularia* are shown in (b). The probabilities were produced by a complementary log‐log (cloglog) transform, which is considered to be given a stronger theoretical justification than the logistic transform (Phillips et al., 2017). The average of replicate runs of the probabilities are shown in color on a per grid

Nine bioclimatic variables remained after removing those that were strongly correlated, namely Bio1: annual mean temperature, Bio2: mean diurnal range, Bio7: temperature annual range, Bio8: temperature of wettest quarter, Bio12: annual precipitation, Bio14: precipitation of driest month, Bio15: precipitation seasonality, Bio18: precipitation of warmest quarter, and Bio19: precipitation of coldest quarter (for further details, see WorldClim website). The ecological niche model of *R*. *plicatula* had an average test AUC value of 0.9742 (Table 4). The ecological niche model of *R. auricularia* also had an average test AUC value of 0.8523 (Table 4). For the ecological niche model of *R*. *plicatula*, Bio14 and Bio1 had the highest and second highest contributions to the predictive model, respectively, and Bio1 and Bio14 had the highest and second highest permutation importance, respectively (Table 4). For the ecological niche model of *R. auricularia*, Bio1 and Bio7 had the highest and second highest contributions to the predictive model, respectively, and Bio1 and Bio 14 had the highest and second highest permutation importance, respectively (Table 4). The three jackknife tests of the ecological niche model of *R*. *plicatula* suggested that Bio18 and Bio14 were relatively important variables (Figure S4.3). The three jackknife tests of the ecological niche model of *R. auricularia* suggested that Bio1 and Bio14 were relatively important variables (Figure S4.3). The occurrence probabilities estimated from current climate variables showed that some environmental factors could restrict the distribution area of *Radix* species (Figure 5).

**TABLE 4:** The training area under the receiver operator curve (AUC) values, the test AUC values, contribution, and permutation importance percentages of each bioclimatic variable of two *Radix* species

	*Radix plicatula*	*Radix auricularia*
Test AUC	0.9742	0.8523
Training AUC	0.9768	0.868
Bio1 contribution	15.5646%	58.9643%
Bio1 permutation importance	48.5994%	58.0762%
Bio2 contribution	12.9102%	0.2651%
Bio2 permutation importance	3.2928%	0.5548%
Bio7 contribution	0.8336%	17.2299%
Bio7 permutation importance	5.01%	0.8412%
Bio8 contribution	14.3721%	0.9798%
Bio8 permutation importance	9.9732%	8.8737%
Bio12 contribution	0.089949%	3.7308%
Bio12 permutation importance	2.8737%	5.8677%
Bio14 contribution	37.1584%	15.5319%
Bio14 permutation importance	21.2927%	18.2921%
Bio15 contribution	0.036%	0.4076%
Bio15 permutation importance	0.5972%	3.9344%
Bio18 contribution	9.8035%	2.8906%
Bio18 permutation importance	6.7464%	3.56%
Bio19 contribution	0.3266%	0%
Bio19 permutation importance	1.6145%	0%

## 4 DISCUSSION

Our geographical reconstruction estimated that the origin of *Radix* species was located around the Indian subcontinent (Figure 4a). This result is consistent with a previous study by Aksenova et al. (2018). In addition, Aksenova et al. (2018) suggested that the first occurrence of *Radix* was around the late Eocene based on reliable fossil calibrations. The temperature around the late Eocene was c.a. 5°C warmer than the current temperature (Hansen et al., 2013; Zachos et al., 2001). Therefore, *Radix* seemingly originated under a tropical climate.

After the origin, the distribution area of *Radix* expanded (Figure 4), and consequently, 11 lineages diversified based on our estimations (Figures 2 and 3 and Figure S4.1). In the family Lymnaeidae, morphological taxonomy often causes confusion, and thus the species delimitation has been detected using molecular taxonomic approaches (Aksenova et al., 2017, 2018; Pfenninger et al., 2006; Puslednik et al., 2009). Based on the previous delimitation on *Radix* (Aksenova et al., 2018), nine species and one undescribed species were detected. The results of our phylogenies were largely consistent with this previous study, although one species, *R*. *onychia*, was phylogenetically confirmed for the first time in our study and monophyly of *R*. *plicatula* and *R. rufescens* was not well supported (BPP < 0.95 and BV < 0.95). Furthermore, our phylogeny showed distinctive subclades within some species (Figure S4.1; A.I–VI, F.I–IV). A further integrative approach (Dayrat, 2005; e.g., Aksenova et al., 2017; Bolotov et al., 2014, 2017; Vinarski et al., 2016) is needed to clarify the actual status of these subclades; however, these lineages could be recognized as evolutionary units.

*Radix auricularia*, which was distributed in the most northern area of the 11 species, had the widest distribution area; *R*. *natalensis*, *R*. *rubiginosa*, and *R*. *plicatula* also had relatively wide distribution areas (Figure 1 and Table 1). In contrast, *R*. *alticola*, *R*. *brevicauda*, *R*. *onychia*, *R*. *makhrovi*, *R. rufescens*, and *Radix* sp. had quite limited distribution areas (Figure 1 and Table 1). Although we were not able to eliminate the possibility of human introduced populations in the selection of *Radix* sequences, the distribution areas and phylogenetic relationship within each species geographically have a certain consistency. Except for two endemic species on ancient lakes (*R*. *onychia* and *Radix* sp.), the distributions of these species were concentrated around the origin of the genus (Figure 1). This geographic pattern seems to be in accord with the LDG under NC, although our model system did not have an adequate number of species to examine the LDG with this taxon. In contrast, the limited distributions in the high diversity area may also suggest interspecific competition (Pontarp et al., 2019). Nevertheless, most of the species had overlapping distribution areas (Figure 2), and the effect of interspecific competition on each distribution may be limited.

Our geographic reconstruction showed distribution transitions throughout the evolutionary history of *Radix* (Figure 4). Most of the colonization routes had a longer immigration distance in the longitudinal direction than in the latitudinal direction (Figure 4 and Table 3). Although there is a topographical bias, for example, in Eurasia, which is the dominant distributed region of *Radix*, there is little difference in distance from easternmost [Cape Dezhnev] to westernmost [Cabo da Roca] and from northernmost [Cape Chelyuskin] to southernmost [Tanjung Piai]. Furthermore, considering the dispersal ability of freshwater mollusks (Kappes & Haase, 2012) and estimated immigration distances (Table 3), the topographic scale of the landmass can be considered as sufficiently large for single colonization. Accordingly, the potential immigration distance in latitudinal and longitudinal directions essentially should have no significant difference. Therefore, this difference would suggest that the colonization to latitudinal direction may be limited by some factors. A lot of factors can limit colonizations such as geographic barriers, habitat type, food availability, the presence of predators, and interspecific competition. Our results cannot eliminate the contribution of these to the evolutionary history of *Radix*; however, most of these factors may be not strongly related to latitudinal and/or longitudinal directions, and so they may be unlikely to be the main factors of difference between latitudinal and longitudinal immigration distance. In contrast, climate, in particular, temperature is strongly correlated with latitude; therefore, the restriction on latitudinal immigration distance indicates that the climate may be a barrier to the colonization of *Radix*. In general, adaptation to temperature is important for the evolution of organisms, and both direct adaptation (e.g., temperature tolerance of *Radix* species) and indirect adaptation (interaction with other organisms adapted to the temperature) can be considered (Barton, 2011; Clarke, 2003). Further researches, especially direct quantification of ecological traits of *Radix*, are needed to determine which mechanism is principal for the evolutionary history of *Radix*. In any case, this indicates that the difficulty in climatic adaptation (i.e., NC) may have determined the distribution transitions in the evolutionary history of *Radix*. Many phylogeographical studies have shown that distribution transitions over climate zones are difficult (e.g., Economo et al., 2018, 2019; Kerkhoff et al., 2014; Kozak & Wiens, 2010; Stephens & Wiens, 2009). In particular, many animal taxa with high active dispersal abilities have been found to have restricted historical transitions in their distribution areas due to climate factors (e.g., Bats: Buckley et al., 2010; Stevens, 2011; Birds: Duchêne & Cardillo, 2015; Hawkins et al., 2006; Butterflies: Hawkins & DeVries, 2009; Owens et al., 2017; Flies: Löwenberg‐Neto et al., 2011). In contrast, few studies have focused on animal taxa with passive dispersals. However, our results showed that climate was also an important factor in determining the distribution of passive dispersers, which have a more probabilistic dispersal mode. In plants, which are critically different from animals in terms of ecology and genetics, many studies have shown the importance of climate to distribution (e.g., Kerkhoff et al., 2014). This may be due to the fact that many plants are passive dispersers. Regardless of the dispersal mode, colonization seems to be restricted by latitudinal climate when the distribution of an organism expands by dispersal.

Furthermore, our ecological niche models showed that the current distribution ranges of *Radix* species were restricted by climate (Figure 5 and Figure S4.3). Based on our modeling, which had a high prediction accuracy despite the limited climate variables used, the annual mean temperature (Bio1) and precipitation of driest month (Bio14) were relatively influential factors for both of the species analyzed under all the modeling approaches used (Table 4 and Figure S4.3). Air temperature is often a critical factor in determining distribution ranges (Calosi et al., 2010; Merriam, 1984), and it is also important for freshwater organisms (Calosi et al., 2010; Cordellier et al., 2012) because shallow water temperature is strongly correlated with air temperature. Moreover, precipitation of the driest month can be an important factor because droughts affect the population dynamics of freshwater mollusks that inhabit temporal inland water (Gérard, 2001; Woolhouse, 1992). The restriction to the distribution ranges, which caused by these climate variables, was clear; however, the potential distribution ranges seemingly had fairly wide (Figure 5). This fits well with the high niche flexibility within the species (Cordellier & Pfenninger, 2009; Torres et al., 2018).

Our analyses showed two suggestions about the evolutionary history of *Radix*: their colonization is more likely to occur in the longitude direction than in the latitude direction, and one of the important determinants of the current *Radix* distribution can be temperature. Considering that temperature is strongly correlated with latitude, the restriction by the temperature has influenced the distribution transitions of *Radix*, either directly or indirectly, and then has established their current distribution pattern, although the intervention of other factors such as interspecific competition cannot be excluded. Thus, the present findings suggest that NC to the climate of habitat, as a mechanism, affected the distribution transitions of *Radix*, which previously had not been clearly shown in freshwater mollusks. Although further studies are needed to clarify the evolutionary patterns of freshwater mollusk niches and dispersal mechanisms, our suggestions may show that distribution transitions over latitudinal climate zones were restricted even on the freshwater mollusks that are mainly passive disperser.

## CONFLICT OF INTEREST

The authors declare that they have no competing interests.

## AUTHOR CONTRIBUTIONS

**Takumi Saito:** Conceptualization (lead); data curation (lead); formal analysis (lead); funding acquisition (equal); investigation (lead); methodology (lead); resources (lead); software (lead); validation (lead); visualization (lead); writing – original draft (lead); writing – review & editing (lead). **Takahiro Hirano:** Data curation (supporting); investigation (equal); resources (supporting); supervision (supporting); validation (equal); writing – review & editing (equal). **Bin Ye:** Data curation (supporting); investigation (supporting); resources (equal); writing – review & editing (equal). **Larisa Prozorova:** Data curation (supporting); investigation (supporting); project administration (supporting); resources (equal); writing – review & editing (equal). **Mohammad Shariar Shovon:** Investigation (supporting); project administration (supporting); resources (equal); writing – review & editing (equal). **Tu Van Do:** Investigation (supporting); project administration (supporting); resources (equal); writing – review & editing (equal). **Kazuki Kimura:** Data curation (supporting); funding acquisition (supporting); investigation (supporting); project administration (supporting); resources (equal); writing – review & editing (equal). **Purevdorj Surenkhorloo:** Project administration (supporting); resources (equal); writing – review & editing (equal). **Yuichi Kameda:** Data curation (supporting); investigation (supporting); resources (supporting); supervision (supporting); writing – review & editing (equal). **Yuta Morii:** Data curation (supporting); investigation (supporting); resources (supporting); writing – review & editing (equal). **Hiroshi Fukuda:** Data curation (supporting); investigation (supporting); resources (supporting); supervision (supporting); writing – review & editing (equal). **Satoshi Chiba:** Conceptualization (supporting); funding acquisition (equal); methodology (supporting); project administration (lead); supervision (lead); writing – review & editing (equal).

## Supporting information

Table S1‐S3

Fig S4

## Data Availability

All obtained sequences were deposited in the GenBank (Accession Nos. LC658376‐LC658655, LC659004‐LC659079, LC659091‐LC659234 and LC663694‐LC663695). Additional data are available from supplementary materials.
