# Supplementary material for: A comprehensive phylogeography of the widespread pond snail genus Radix revealed restricted colonization due to niche conservatism
Source: Ecol Evol. 2021 Dec 17;11(24):18446–59. doi: 10.1002/ece3.8434 (PMC8717273; doi:10.1002/ece3.8434)

Figure S4.1

The Bayesian phylogenetic tree of genus *Radix* inferred from 455 CO1 haplotypes from 750 sequences (660 bp). Eight species were selected as outgroups. Scale bar indicates substitutions per site. Each operational taxonomic unit label represents the haplotype number in Table S1. Numbers on the branches indicate the Bayesian posterior probabilities (BPP) and the Maximum likelihood ultrafast bootstrapping value by IQ-TREE (Nguyen, Schmidt, von Haeseler, & Minh, 2015; Hoang, Chernomor, von Haeseler, Minh, & Vinh, 2017). These values of low supported (BPP < .070) and terminal branches are not shown. Vertical bars on the right side indicate species and nominal clades within each species. Each Radix species were determined based on previous study (Aksenova et al. 2018)

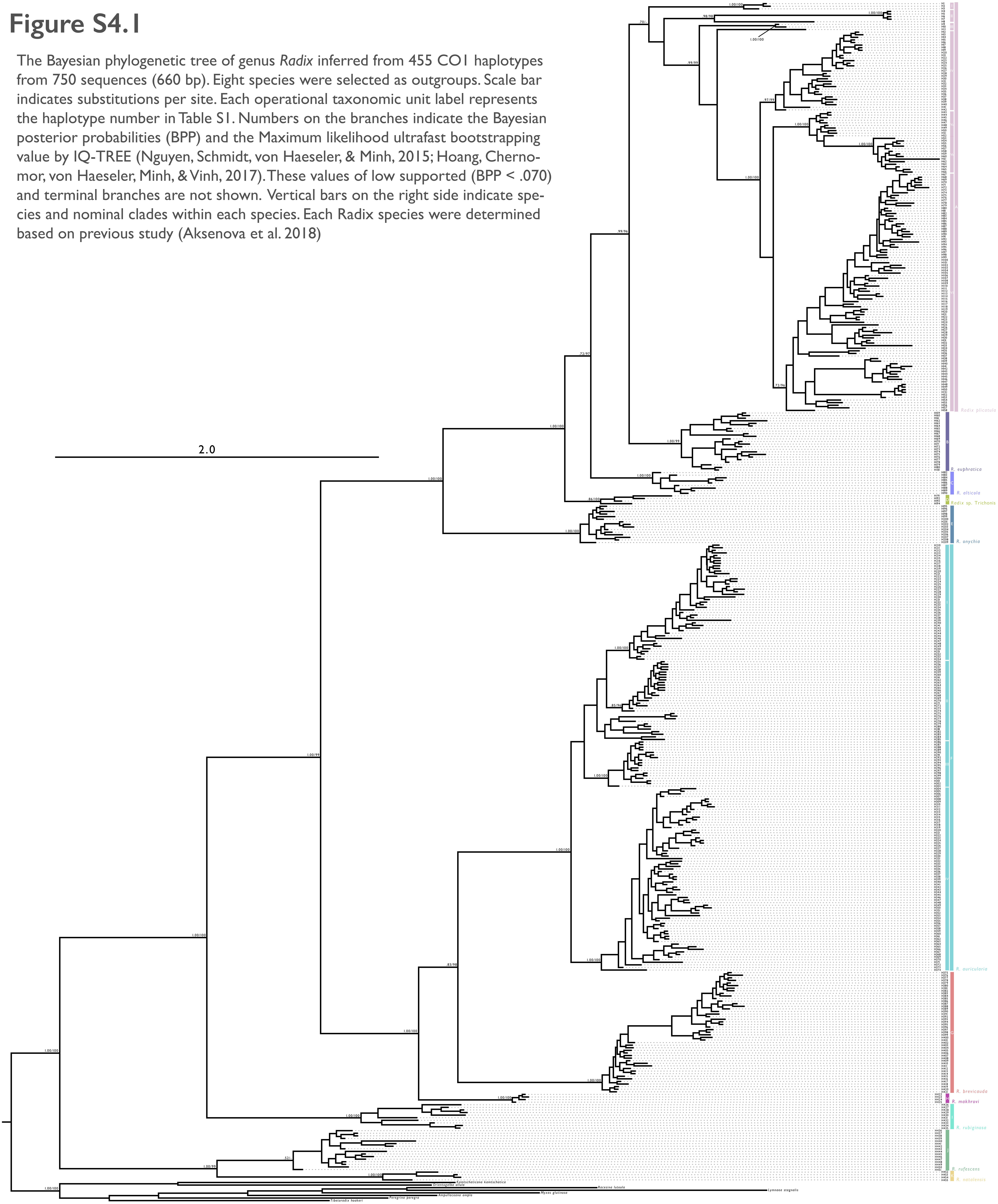

Figure S4.2

The Bayesian phylogenetic tree of genus *Radix* inferred from nuclear dataset (1285 bp). *Racesina luteola* was selected as outgroup. Each operational taxonomic unit label represents the material ID. Scale bar indicates substitutions per site. Numbers on the branches indicate the Bayesian posterior probabilities (BPP) and the Maximum likelihood ultrafast bootstrapping value by IQ-TREE (Nguyen, Schmidt, von Haeseler, & Minh, 2015; Hoang, Chernomor, von Haeseler, Minh, & Vinh, 2017). These values of terminal branches are not shown. See Table S1.1 for further information

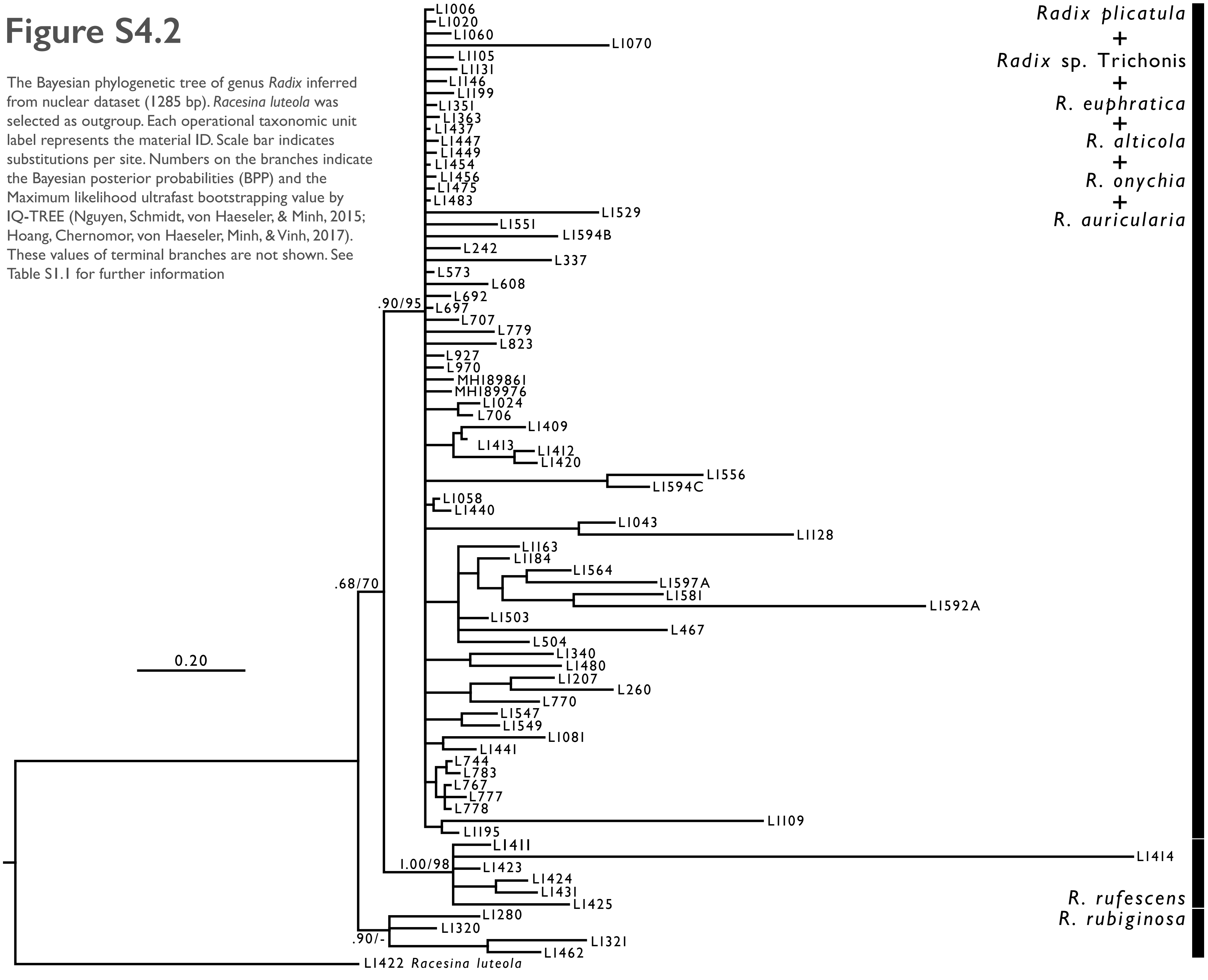

Figure S4.3

The values with all variables

The values without each variable

The values with only each variable

The three different types of jackknife test. Tests for *Radix plicatula* are shown in a to c, and tests for *R. auricularia* are shown in d to f. Purple bars represent the values obtained with all variables. Sky blue bars represent the values obtained without each variable. Dark blue bars represent the values obtained with only each variables. All values are averages over all replicates run. Each variables show below: Bio1; annual mean temperature, Bio2; mean diurnal range, Bio7; temperature annual range, Bio8; temperature of wettest quarter, Bio12; annual precipitation, Bio14; precipitation of driest month, Bio15; precipitation seasonality, Bio18; precipitation of warmest quarter, Bio19; precipitation of coldest quarter.

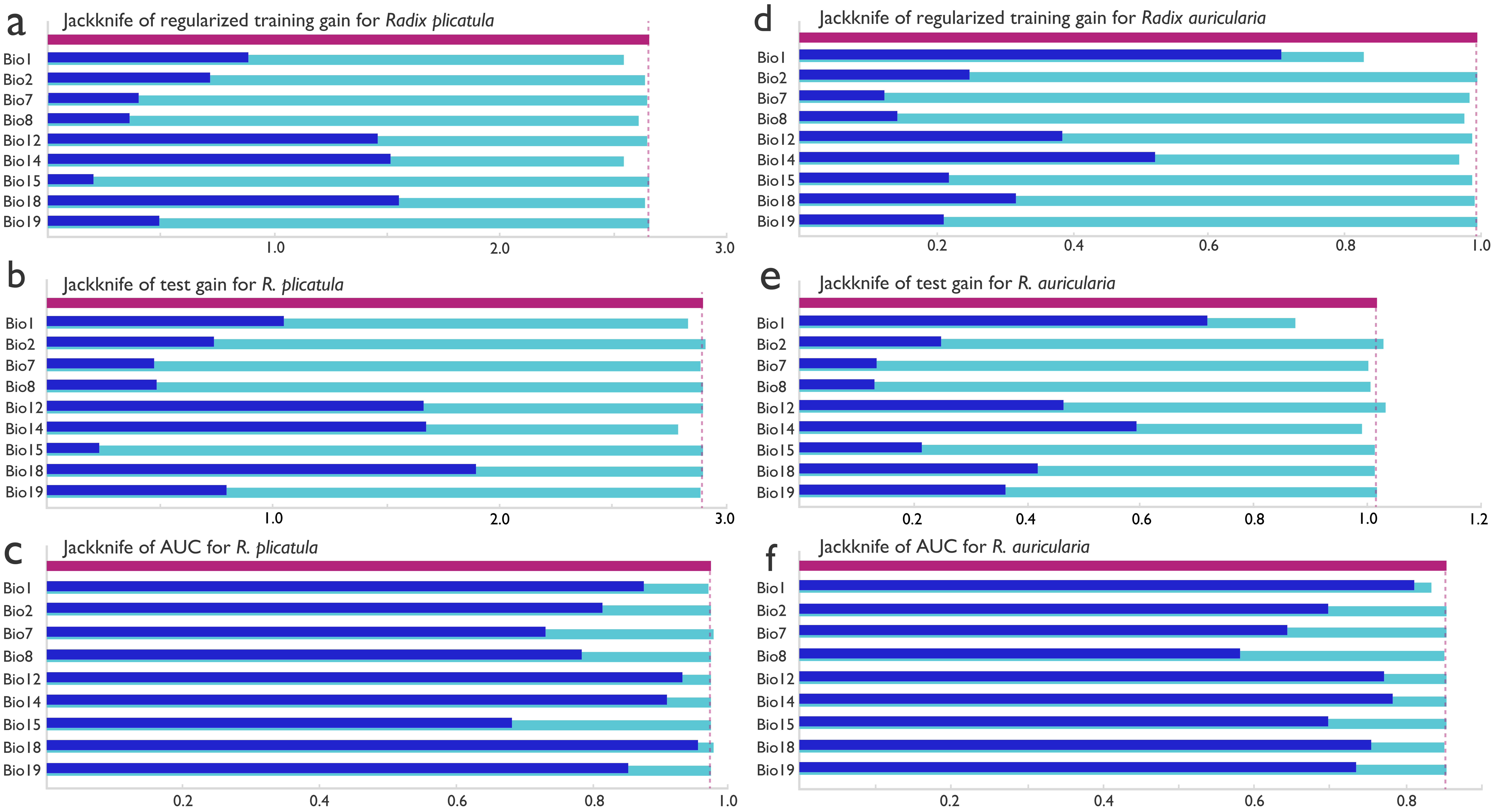

Supplement: Supplementary file 2 — Fig S4 [file ECE3-11-18446-s001.pdf]
